# Prediction model for severe maternal morbidity in pregnant women with hypertensive disorders of pregnancy

**DOI:** 10.12669/pjms.40.6.9095

**Published:** 2024-07

**Authors:** Yan Bian, Shuisen Zheng, Xiuwu Liu, Xiuming Jiang

**Affiliations:** 1 Yan Bian, Labour Ward, Fujian Maternity and Child Health Hospital College of Clinical Medicine for Obstetrics & Gynecology and Pediatrics, Fujian Medical University,Fuzhou City, Fujian Province, China; 2 Shuisen Zheng, Department of of Obstetrics Resident Physician. Fujian Maternity and Child Health Hospital College of Clinical Medicine for Obstetrics & Gynecology and Pediatrics, Fujian Medical University,Fuzhou City, Fujian Province, China; 3 Xiuwu Liu, Department of Nursing, Labour Ward, Fujian Maternity and Child Health Hospital College of Clinical Medicine for Obstetrics & Gynecology and Pediatrics, Fujian Medical University,Fuzhou City, Fujian Province, China; 4 Xiuming Jiang, Fujian Maternity and Child Health Hospital College of Clinical Medicine for Obstetrics & Gynecology and Pediatrics, Fujian Medical University,Fuzhou City, Fujian Province, China

**Keywords:** Hypertensive disorders of pregnancy, Multivariate logistic regression analysis, Nomogram, Concordance index, Severe maternal morbidity

## Abstract

**Objectives:**

To investigate risk factors for severe maternal morbidity (SMM) in pregnant women with hypertensive disorders of pregnancy (HDP) and to develop a risk prediction model.

**Methods:**

A prospective observational cohort study was conducted among pregnant women who were hospitalized for hypertensive disorders of pregnancy (HDP) between January 2016 and December 2020 in Fujian College of Clinical Medicine for Obstetrics & Gynecology and Pediatrics, Fujian Province, China (a training set), and a risk predictive model was constructed. Pregnant women with HDP who were hospitalized between January 2021 and December 2021 were selected as a validation set. Concordance index (C-index) and calibration curves were used to test predictive model discrimination and calibration.

**Results:**

We included 970 pregnant women (790 in the training set and 180 in the validation set). Least absolute shrinkage and selection operator regression was used to screen for nine related variables such as intra-uterine growth retardation (IUGR), diastolic blood pressure (DBP) and systolic blood pressure (SBP) at suspected diagnosis, total bilirubin, albumin (ALB), uric acid, total cholesterol, serum magnesium, and suspected gestational age. SBP at suspected diagnosis (OR =1.22, 95%CI:1.08–1.42) and total cholesterol (OR = 1.78, 95%CI:1.17–2.80) were independent risk factors of severe maternal morbidity in pregnant women with HDP. A nomogram was constructed, and internal validation of the nomogram model was done using the bootstrap self-sampling method. C-index in the training and the validation set was 0.798 and 0.909, respectively.

**Conclusion:**

Our prediction model can be used to determine gestational hypertension severity in pregnant women.

**Abbreviations:**
SMM: Severe maternal morbidity;
HDP: Hypertensive disorders of pregnancy;
LASSO: Least absolute shrinkage and selection operator;
IUGR: intrauterine growth retardation;
SBP: Systolic blood pressure;
DBP: Diastolic blood pressure;
ALB: Serum albumin concentration;
URIC: Serum uric acid concentrations.

## INTRODUCTION

Severe maternal morbidity (SMM) is an unexpected labor outcome that leads to significant short and long-term effects on a woman’s health.^1^ Reducing SMM is crucial for reducing maternal mortality and can serve as an important indicator for evaluating the quality of obstetrics. The main causes of SMM are major blood loss, severe sepsis, and preeclampsia-associated conditions.^2^ Hypertensive disorders of pregnancy (HDP) are associated with high rate of maternal and fetal mortality.^3^ Studies show that the most common SMM types are Hemolysis, Elevated Liver enzymes and Low Platelets (HELLP) syndrome and severe preeclampsia, with the incidence of 514.6 per 100,000 pregnancies.^4^ Adequate and timely screening of high-risk pregnant women who are prone to SMM in the early stage of HDP became a topic of considerable research interest due to the high risk of SMM in women with gestational hypertension disorder. This prospective cohort study aimed to detect and analyze clinical characteristics and related risk factors of SMM in pregnant women with HDP from the onset of HDP to 42 days after delivery. We established a SMM clinical risk-prediction model in pregnant women with HDP and evaluated its accuracy.

## METHODS

This prospective cohort study included 970 women with singleton pregnancies who were hospitalized for HDP at the Fujian College of Clinical Medicine for Obstetrics & Gynecology and Pediatrics. Patients were prospectively enrolled in the study between January 2016 and December 2020. According to the timing of the included studies, pregnant women hospitalized between January 2016 and December 2020 were used as the training set (790 cases), and pregnant women hospitalized between January 2021 and December 2021 were used as the validation set (180 cases).

### Inclusion criteria:

- Age>18 years.
- Diagnosis of HDP.^5^
- Diagnosis of SMM.^1^
- Complete medical records.

### Exclusion criteria:

- Pregnant women with SMM before admission.
- Women with missing data.
- Patients with pre-existing conditions such as chronic kidney disease, thrombocytopenia, lupus nephritis, coagulopathy.

### Diagnostic criteria

SMM was diagnosed based on the criteria of the 2016 American College of Obstetricians and Gynecologists (ACOG) and the Society for Maternal Fetal Medicine (SMFM) guidlines.^1^ Briefly, two criteria were used to screen for severe maternal morbidity: 1) transfusion of four or more units of blood and 2) admission of a pregnant or postpartum woman to an ICU.

HDP was diagnosed based on the guidelines of HDP (2020) issued by the Chinese Medical Association Obstetrics and Gynecology Branch,^5^ and divided into four subtypes: gestational hypertension, preeclampsia-eclampsia, chronic hypertension, and chronic hypertension with superimposed preeclampsia.

### Data collection

Clinical data were collected according to the standard operating procedure from patients upon admission (with suspected blood pressure elevation, positive urine protein, or signs of labor) and included general information, such as age, body mass index (BMI), heart rate (HR), blood pressure (BP), and laboratory indicators on admission day, such as liver function indices, routine blood tests, renal function, blood lipid parameters, coagulation function, and coagulation function (Table-I and II).

**Table-I:** Clinical characteristics of the training and test cohort.

Variables	Total (n = 970)	Test cohort (n = 180)	Train cohort (n = 790)	P value
Age (y)	30 [27, 34]	29 [26, 33]	30 [27, 34]	0.031
Age >35				0.069
No	786 (81)	155 (86)	631 (80)	
Yes	184 (19)	25 (14)	159 (20)	
GDM (n (%))				0.528
No	712 (73)	136 (76)	576 (73)	
Yes	258 (27)	44 (24)	214 (27)	
Twins pregnancy (n (%))				0.406
No	870 (90)	165 (92)	705 (89)	
Yes	100 (10)	15 (8)	85 (11)	
ICP (n (%))				1
No	952 (98)	177 (98)	775 (98)	
Yes	18 (2)	3 (2)	15 (2)	
Scarred uterus (n (%))				0.134
No	795 (82)	155 (86)	640 (81)	
Yes	175 (18)	25 (14)	150 (19)	
IUGR (n (%))				0.142
No	874 (90)	168 (93)	706 (89)	
Yes	96 (10)	12 (7)	84 (11)	
BMI ≥28 (n (%))				0.169
No	543 (56)	92 (51)	451 (57)	
Yes	427 (44)	88 (49)	339 (43)	
SMM (n (%))				0.306
No	854 (88)	163 (91)	691 (87)	
Yes	116 (12)	17 (9)	99 (13)	
PROM (n (%))				0.7
No	763 (79)	144 (80)	619 (78)	
Yes	207 (21)	36 (20)	171 (22)	
Placental abruption (n (%))				1
No	939 (97)	174 (97)	765 (97)	
Yes	31 (3)	6 (3)	25 (3)	
Fetal distress (n (%))				0.598
No	865 (89)	163 (91)	702 (89)	
Yes	105 (11)	17 (9)	88 (11)	
Placenta previa (n (%))				1
No	963 (99)	179 (99)	784 (99)	
Yes	7 (1)	1 (1)	6 (1)	
SBP (mmHg)	136 (128, 141)	136 (127, 140.25)	136 (128.25, 141)	0.519
DBP (mmHg)	85 (79, 89)	85 (79, 89)	85 (79, 89)	0.993
Heart rate (times/min)	88 (82, 95)	88 (82, 95)	88 (81, 95)	0.707
Gestational week	38.71 (36.86, 39.71)	38.64 (36.21, 40.14)	38.71 (36.86, 39.71)	0.966

***Abbreviations:*** GDM, gestational diabetes; ICP, intrahepatic cholestasis of pregnancy; IUGR, intrauterine growth retardation; SMM, severe maternal morbidity; PROM, premature rupture of membranes; SBP, systolic blood pressure; DBP, diastolic blood pressure.

**Table-II:** General baseline information.

		Non-SMM group	SMM group	P Value
Age (y)		30.39 (5.14)	32.13 (5.22)	0.001
Gestational week		38.86 [37.00, 39.86]	37.28 [34.43, 38.78]	<0.001
GDM (n (%))	NO	630 (73.8)	82 (70.7)	0.553
	YES	224 (26.2)	34 (29.3)	
Twins pregnancy (n (%))	NO	769 (90.0)	101 (87.1)	0.408
	YES	85 (10.0)	15 (12.9)	
ICP (n (%))	NO	838 (98.1)	114 (98.3)	1
	YES	16 (1.9)	2 (1.7)	
Scarred uterus (n (%))	NO	711 (83.3)	84 (72.4)	0.007
	YES	143 (16.7)	32 (27.6)	
IUGR (n (%))	NO	783 (91.7)	91 (78.4)	<0.001
	YES	71 (8.3)	25 (21.6)	
BMI (n (%))	< 28	488 (57.1)	55 (47.4)	0.06
	≥ 28	366 (42.9)	61 (52.6)	
PROM (n (%))	NO	665 (77.9)	98 (84.5)	0.131
	YES	189 (22.1)	18 (15.5)	
placental abruption (n (%))	NO	832 (97.4)	107 (92.2)	0.007
	YES	22 (2.6)	9 (7.8)	
fetal distress (n (%))	NO	762 (89.2)	103 (88.8)	1
	YES	92 (10.8)	13 (11.2)	
placenta previa (n (%))	NO	847 (99.2)	116 (100.0)	0.694
	YES	7 (0.8)	0 (0.0)	
SBP (mmHg)		135.00 [128.00, 140.00]	150.00 [137.75, 154.00]	<0.001
DBP (mmHg)		85.00 [79.00, 88.00]	92.00 [84.75, 96.00]	<0.001
Heart rate(times/min)		88.00 [82.00, 95.00]	87.50 [80.00, 93.25]	0.253
WBC (×10^9^)		9.35 [7.88, 11.58]	9.55 [7.98, 11.80]	0.470
hemoglobin (g/L)		119.13±15.39	120.37±17.94	0.424
PLT (×10^9^)		207.18±58.48	196.53±65.30	0.070
MPV (fL)		11.00 [10.30, 11.80]	11.30 [10.50, 11.90]	0.063
PDW (fL)		13.00 [11.50, 14.80]	13.70 [11.67, 15.90]	0.072
ANC (×10^9^)		6.90 [5.53, 9.00]	6.85 [5.30, 9.30]	0.706
HCT (%)		35.50 [32.70, 37.90]	35.60 [32.60, 38.38]	0.272
LY (×10^9^)		1.65 [1.34, 1.99]	1.71 [1.33, 2.15]	0.270
PLCR (%)		33.29±9.01	34.13±8.76	0.345
PT (s)		11.08±0.76	10.84±0.82	0.001
FDP (mg/L)		6.20 [3.80, 9.80]	6.30 [3.80, 9.90]	0.687
PTA		111.20 [102.20, 124.10]	116.10 [106.60, 136.10]	0.003
Fib (g/L)		4.05 [3.56, 4.62]	3.98 [3.48, 4.54]	0.241
D-dimer (mg/LFEU)		1.98 [1.22, 3.41]	1.89 [1.16, 3.80]	0.899
URIC (mmol/L)		340.80 [283.00, 406.80]	366.00 [304.90, 452.95]	0.006
ALT (U/L)		14.60 [9.70, 21.00]	16.00 [10.65, 28.50]	0.010
AST (U/L)		20.00 [15.60, 25.00]	22.15 [17.10, 28.33]	0.004
GGT (U/L)		12.00 [9.60, 17.50]	12.00 [10.00, 19.00]	0.403
ALP (U/L)		156.70 [124.53, 199.88]	144.05 [112.12, 202.25]	0.218
ALB (g/L)		32.90 [30.60, 34.70]	31.10 [28.20, 33.12]	<0.001
TBIL (umol/L)		8.50 [6.60, 10.70]	8.50 [6.47, 11.03]	0.918
DBIL (umol/L)		1.45 [0.00, 2.00]	1.30 [0.00, 1.80]	0.041
IBIL (umol/L)		6.30 [5.00, 8.20]	6.75 [5.18, 8.53]	0.269
Triglycerides (mmol/L)		3.50 [2.70, 4.60]	3.50 [2.70, 4.73]	0.660
CHOL (mmol/L)		5.90 [5.10, 6.90]	6.50 [5.50, 7.55]	<0.001
HDL (mmol/L)		1.80 [1.60, 2.20]	1.80 [1.60, 2.30]	0.365
LDL (mmol/L)		2.80 [2.20, 3.50]	3.20 [2.58, 3.82]	0.004
LDH (U/L)		212.60 [177.17, 465.75]	304.45 [207.62, 544.00]	<0.001
Ca (mmol/L)		2.20 [2.10, 2.30]	2.10 [2.00, 2.20]	<0.001
Mg (mmol/L)		0.80 [0.75, 0.87]	0.86 [0.77, 1.01]	<0.001
BUN (mmol/L)		3.71 [3.07, 4.72]	4.16 [3.25, 5.33]	0.002
Crem (umol/L)		51.60 [45.40, 59.80]	56.15 [46.20, 65.88]	0.005

***Abbreviations:*** GDM, gestational diabetes; ICP, intrahepatic cholestasis of pregnancy; IUGR, selective intrauterine growth restriction; SMM, severe maternal morbidity; PROM, premature rupture of membranes; SBP, systolic blood pressure; DBP, diastolic blood pressure; ALT, alanine aminotransferase; AST, aspartate aminotransferase; CREm, creatinine; FDB, fibrinogen degradation product; Fib, fibrinogen; GGT, transglutaminase; HDL, high-density lipoprotein; LDL, low-density lipoprotein; INR, international normalized ratio; LDH, lactate dehydrogenase; MPV, mean platelet volume; PT, prothrombin time; TG, triglycerides; URIC, uric acid; TBIL, total bilirubin; DBIL, direct bilirubin; IBIL, indirect bilirubin; CHOL, cholesterol; ALB, albumin.

Maternal and neonatal complications, such as placenta previa, placental abruption, gestational diabetes (GDM), scarred uterus, premature rupture of membranes (PROM), twin pregnancy, intrahepatic cholestasis of pregnancy (ICP), fetal distress and intrauterine growth retardation (IUGR) were recorded (Table-I and II). Patients who gave birth or were discharged for other reasons had their contact details recorded and were informed about the follow-up plan through regular outpatient and telephone follow-ups.

### Sample size calculation

The training set underwent sample size calculation based on the events per variable (EPV) rule, which suggests 10-15 cases per explanatory variable. Therefore, this study anticipated including 10 predictor variables, indicating that 100 cases (10×10) would have been needed for the analysis to prevent model overfitting. A total of 116 patients in the training set of this study had SMM, thereby meeting the sample size requirements for modeling.

### Statistical analysis

Statistical analysis was done using the R (V3.6.2). Measurement data were presented as median (interquartile range [IQR]) or mean ± standard deviation (X±SD) and compared between both groups using non-parametric or t-tests, as appropriate. Enumeration data were expressed as n (%) and were analyzed using the chi-squared test, corrected χ^2^ test, or Fisher’s exact probability method, as required. Least absolute shrinkage and selection operator (LASSO) regression was used to screen high-risk factors in the training set data with 1SE as the benchmark. To fit the model, multivariate logistic regression was used, followed by the odds ratio (OR) and 95% confidence interval (CI), as well as nomograms to visualize the model. The training and the validation sets were used to validate the predictive ability of the nomogram models, respectively. The results were measured using the index of concordance (C-index) and the calibration curve was used to measure the prediction accuracy. P-values of<0.05 indicated statistical significance. The analyses were performed using R packages “MASS”, “rms”, “nnet”, “Hmisc”, etc.

### Ethics committee approval

Ethics Committee of Fujian Maternity and Child Health Hospital approved this study (IRB-2020Y183 dated Dec. 20, 2022) and authorized waiving written informed patient consent because there were no interventions for patient care at any stage of the research. The study was conducted in accord with the guidelines of the Declaration of Helsinki, and the rights of all participants were protected. Permission to access anonymized (non-identified) data was granted by the Fujian Maternity and Child Health Hospital Database Steering Committee.

## RESULTS

### Baseline characteristics

**Fig.1:**
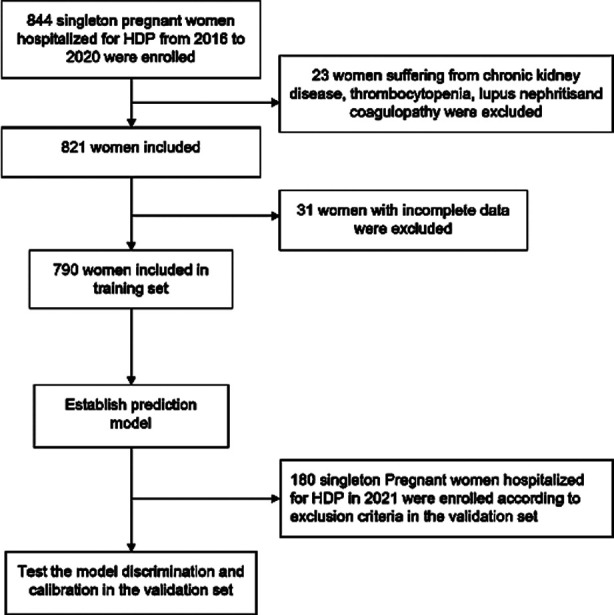
Flowchart of the enrolled patients.

A total of 970 pregnant women were included in the study. Of them, 790 were hospitalized between January 2016 and December 2020 and served as the training set, while 180 were hospitalized between January 2021 and December 2021 and served as a validation set (Fig.1). The baseline characteristics did not differ significantly between the training and the validation sets (Table-I). Similarly, rates of comorbid disorders did not differ between the groups. Age, BMI, incidence of GDM, ICP, PROM, twin pregnancy, scarred uterus, IUGR, placenta previa, placental abruption, fetal distress, blood pressure, or heart rate did not differ in the two groups (Table-I).

**Fig.2A:**
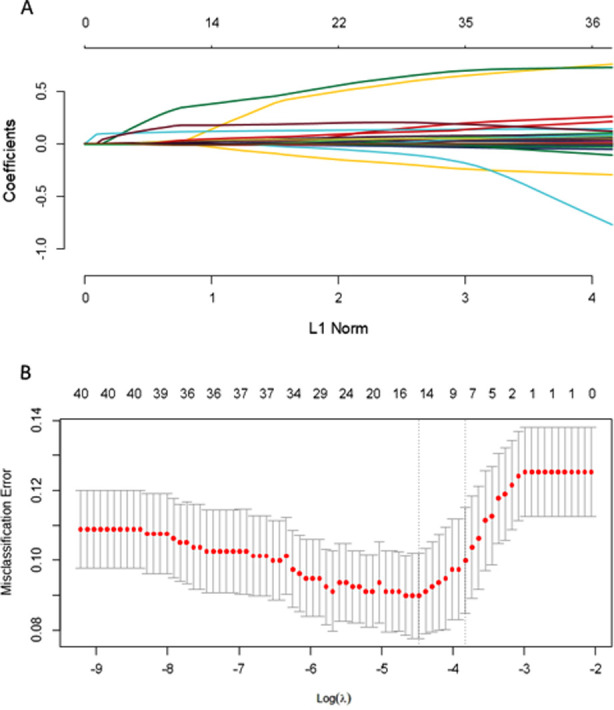
Tuning parameter (lambda) selection in the LASSO model using 10-fold cross-validation via 1SE criteria for determining the risk of SMM in HDP. Fig.2B: Tuning parameter (lambda) selection in the LASSO model using10-fold cross-validation via 1SE criteria for determining the risk of SMM in HDP

There was no statistical difference between the SMM and non-SMM groups in terms of BMI, GDM, twin pregnancy, ICP, PROM, premature placenta, fetal distress, and placenta previa rates (Table-II). Pregnant women in the SMM group were older and had lower suspected gestational age than those in the non-SMM group (P<0.005). The proportion of pregnant women with IUGR was greater in the SMM group than in the non-SMM group (P<0.001). Blood pressure, heart rate, and laboratory test results were compared between the two groups at the time of suspected diagnosis. There were no statistically significant differences in the heart rate between the two groups. The SMM group had significantly higher systolic and diastolic blood pressures than the non-SMM group (P<0.001). In both groups, there were no significant differences in the routine blood test indices, and no significant difference in blood coagulation indices such as fibrinogen degradation product (FDP), D-dimer, and fibrinogen (Fib). The Prothrombin Time (PT) in the SMM group was significantly lower than that in the non-SMM group, and the difference in prothrombin time activity (PTA) levels between the two groups was also statistically significant (P=0.003). Levels of uric acid (URIC), alanine aminotransferase (ALT), aspartate aminotransferase (AST), cholesterol (CHOL), low density lipoprotein (LDL), lactate dehydrogenase (LDH), magnesium (Mg), blood urea nitrogen (BUN), and creatinine (CREm) in the SMM group were significantly higher compared with those in the non-SMM group (P<0.05), whereas albumin (ALB), direct bilirubin (DBIL), and calcium (Ca) were lower compared to the non-SMM group (P<0.001) (Table-II, Fig.2).

### Identification of risk factors for severe maternal morbidity

**Fig.3:**
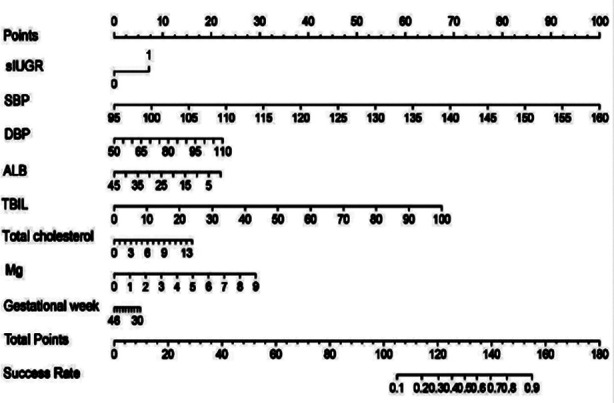
Nomogram for predicting the risk of SMM in HDP.

Subsequently, One Standard Error (1SE) criteria were screened before LASSO regression vetted the variables in the validation set. Eight high-risk factors (IUGR, systolic blood pressure (SBP) and diastolic blood pressure (DBP) at suspected diagnosis, total bilirubin, albumin (ALB), URIC, total cholesterol, Mg, and suspected gestational age) were incorporated in the statistical model (Fig.3). The multi-factor logistic regression analysis showed that SBP (OR1.22, 95%CI:1.08–1.42, P = 0.003) and total cholesterol (OR 1.78, 95%CI:1.17–2.80, P = 0.008) were statistically significant (Table-III). This suggests that pregnant women with gestational hypertension disorders who have higher SBP and total cholesterol values at the time of suspected diagnosis are at a higher risk of adverse maternal outcomes.

**Table-III:** Multivariate logistic regression analysis of variables to identify factors predictive of SMM in Preeclampsia.

Co-variable	Odds ration	95% CI of OR		P-value

		Low Limit	Upper limit	
IUGR	0.31	0.01	3.40	0.403
DBP	1.10	1.00	1.23	0.075
SBP	1.22	1.08	1.42	0.003
ALB	1.00	0.84	1.19	0.990
TBIL	1.06	0.86	1.23	0.542
CHOL	1.78	1.17	2.80	0.008
Mg	1.79	0.67	4.71	0.151
Gestational week	0.84	0.66	1.06	0.137

***Abbreviations:*** CI, confidence interval; DBP, diastolic blood pressure when PE was suspected; SBP, systolic blood pressure when PE was suspected; ALB, albumin; TBIL, total bilirubin; CHOL, total cholesterol; Mg, magnesium

### Evaluation of the predictive model

**Fig.4:**
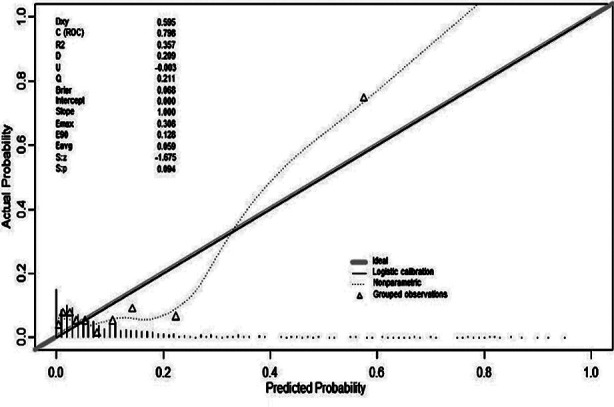
Calibration curves for predicting the risk of SMM in HDP in the training set - nomogram construction (bootstrap = 1 000 repetitions).

**Fig.5:**
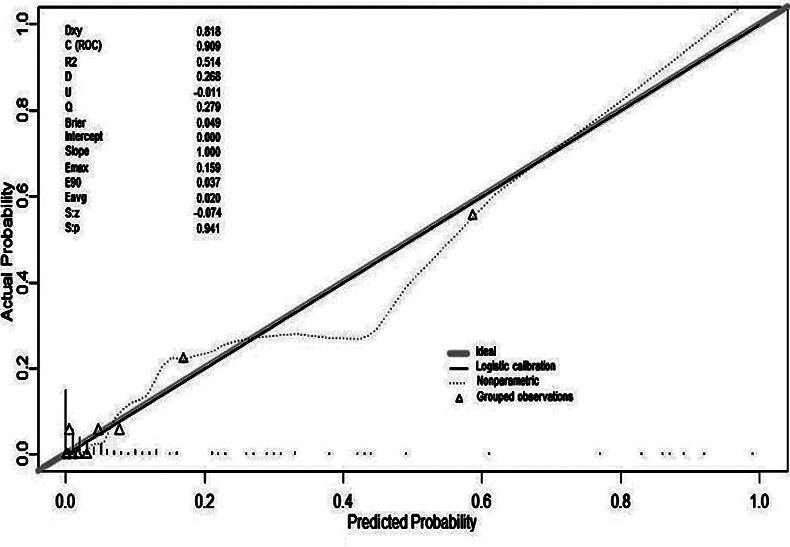
Calibration curves for predicting the risk of SMM in HDP in the validation set - nomogram construction (bootstrap = 1 000 repetitions).

After 1000 bootstrap self-sampling internal validations of the model in the training set, the obtained C-index was 0.798, indicating that the prediction model had a high level of conformity. Additionally, the obtained C-index was 0.909 in the validation set. The calibration curves (Fig.4 and 5) showed that the model’s calibration curve was close to the reference line, which is suggestive of a good calibration.

## DISCUSSION

Our study identified risk factors, associated with SMM in a prospective cohort of pregnant women with HDD. The multivariate logistic regression analysis showed that the abnormal systolic blood pressure and cholesterol levels remained significantly associated with a higher risk of SMM in pregnant women with HDP and were used for creating and validating a prediction model that can be used to determine the severity of gestational hypertension.

Women with HDP have significantly higher probability of adverse maternal and fetal outcomes than normal pregnant women.^6^ Stratified management of maternal risk factors can help to identify high-risk pregnant women in a timely manner and ease the socioeconomic burden on the patients and on the healthcare system.

Currently, SMM is considered the most important indicator of maternal management quality.^7^ According to the current guidelines, SMM is defined as a critical state where woman survives by chance or because of a good care.^8^ Therefore, early identification of potential SMM in pregnant women is crucial.

Pregnant women with preeclampsia who are admitted to the hospital within 48 hours of being diagnosed with the condition need to be monitored for the risk of fatal or life-threatening complications. A prospective, multicenter study by von Dadelszen P et al. developed and validated the fullPIERS model in 2011.^9^ However, this study was limited to high-income European countries. To improve this model, the miniPIERS risk prediction model was subsequently developed for the low- and middle- income countries in 2014.^10^ However, both studies did not include East Asian population. Given the proposed definition of SMM and recent changes in related diagnostic criteria, fullPIERS and miniPIERS models may not be suitable for all settings, particularly primary care settings in East Asian population, and new prediction model for HDP pregnancy women that considers newly introduced concept of SMM is needed.

The American National Partnership for Maternal Safety has developed an effective early warning system based on maternal blood pressure, pulse rate, heart rate, and other vital signs to reduce preventable SMM and maternal mortality. Early identification and treatment of these symptoms can improve maternal outcomes by avoiding critical maternal events.^11^ Additionally, Shields et al. developed a maternal early warning trigger tool (MEWT) for four common maternal comorbidities such as infection, cardiorespiratory insufficiency, preeclampsia-hypertension, and hemorrhage that significantly reduced SMM,^12^ that was shown to reduce SMM and the combined prevalence by 18.4% and 13.6%, respectively.^13^ Our study aimed to build a model that could predict SMM in pregnant women with HDP in the Asian population of southeastern China by using HDP cohort of Fujian Province, China.

Previous approaches, used to predict SMM, were simple and feasible, and most of them relied on changes in maternal vital signs and observations made by bedside nurses. Objective indicators, such as test results, were not included. Although the generalizability of these indexes is good, they often lack accuracy and do not apply to pregnant women hospitalized for HDP. Therefore, we developed our SMM clinical risk prediction model in the prospective cohort of HDP-hospitalized patients and included the occurrence of serious complications from the start of preeclampsia to 42 days after the delivery. The prediction model used IUGR (ultrasound diagnosis), DBP at suspected diagnosis, SBP at suspected diagnosis, total bilirubin, ALB, URIC, total cholesterol, blood magnesium, and suspected gestational age with a high level of accuracy and specificity. We reported improved discrimination in the year following validation. We may speculate that this result relates to the fact that our observational study was not blinded. Therefore, when the prediction model results suggested a high risk of SMM, clinicians strengthened the monitoring of such high-risk groups and found early signs of SMM.

In an observational cohort, Heerden et al. found that blood pressure was an independent risk factor for SMM, such as eclampsia and pulmonary edema in pregnant women with preeclampsia.^14^ After adjusting for confounding factors, we also found that the SBP at the time of suspected diagnosis was a high-risk factor for SMM in pregnant women with HDP (OR =1.22, 95%CI:1.08–1.42, P = 0.003). Changes in blood pressure can affect the perfusion of the placenta and the vital organs. Therefore, it is important to complete ambulatory blood pressure examination in cases of blood pressure that is borderline or has reached the diagnostic criteria for hypertension. That will allow to understand the fluctuation level of blood pressure and to use antihypertensive drugs in a timely manner. High blood pressure that is poorly controlled with medication may indicate disease progression. According to research, blood lipid levels are a distinct risk factor for preeclampsia. Therefore, preeclampsia prediction model may have a high clinical application value.^15^,^16^ However, it is rarely used in predicting SMM in pregnant women. Our study showed that total cholesterol level was a high-risk factor for serious complications in pregnant women with HDP. In a case-control study, Olalere et al. discovered that total cholesterol levels were significantly higher in pregnant women with severe preeclampsia.^17^ Studies show that cholesterol level exceeding 95th percentile can affect fetal blood vessels, leading to poor maternal and neonatal outcomes.^18^ The pathophysiological explanation may be that increased levels of circulating lipids lead to their accumulation in the endothelial cells, reducing prostacyclin release and causing oxidative stress.^19^

Although our study showed that IUGR, DBP at suspected diagnosis, total bilirubin, ALB, URIC, serum magnesium, and suspected gestational age were not independent risk factors for serious complications in HDP pregnant women, their use in the analysis of the predictors, resulted in a model with a high accuracy and specificity. Wu et al. discovered that pregnant women with IUGR were more likely to develop severe preeclampsia, leading to poor maternal and infant outcomes.^20^ Serum albumin can reduce oxidative stress and adverse outcomes in preeclampsia by inhibiting NADPH oxidase activity in human vascular smooth muscle.^21^ Additionally, it has been discovered that serum uric acid levels are significantly elevated in pregnant women with severe eclampsia and these levels are associated with the severity of maternal syndrome and fetal mortality.^22^,^23^until today, the role of uric acid in the clinical course of severe preeclampsia has not been elucidated. Some recent studies suggest that at the time of presentation, subjects with severe preeclampsia frequently have significantly elevated serum uric acid levels, and that the degree of elevation correlates with the severity of the maternal syndrome and fetal morbimortality. In this chapter, we present our workgroup experience. In 2016, we designed a prospective, cross-sectional comparative study. A sample of 200 patients - 100 with severe preeclampsia and 100 with normotensive pregnancy - was obtained. Plasmatic uric acid levels were recorded in units of mg/ dL as clinical variables and as laboratory and fetal growth data. We considered uric acid equal to or more than 6.0 mg/dL as the elevated level. To relate the significance of elevated uric acid levels with variables, chi-square tests and Mann-Whitney U test were applied. Any p value equal or <0.05 was accepted as significant. We found significant difference (p = 0.05 In a multicenter cohort study, Sun F et al. discovered that the total bilirubin level was an independent risk factor for adverse outcomes in pregnant women with preeclampsia.^24^ Early onset preeclampsia is associated with a higher incidence of adverse maternal and infant outcomes compared to late-onset preeclampsia.^25^2003-2008 (n = 456,668 Therefore, the suspected gestational age can be used as an important indicator to be used in the construction of the prediction model.

The higher c-index of the validation set compared to the training set implies that our model has a strong generalization capability. This suggests that our model effectively captures patterns in training data, enhancing predictive accuracy on unseen data. While this result is promising, variations within datasets or unique features in the validation set may also contribute to the improved predictions.

This study was a single-center prospective observational cohort study, and as a regional tertiary hospital in southeastern China, we have accepted referrals from the surrounding areas. The cases are representative, and the results are of reference value.

### Limitations of the study

This is a small sample size study which may potentially impact the reliability of our conclusions, since smaller sample size lowers the p-value.^26^ Further multicenter studies with external validation are needed to verify the stability of the model and expand the research results.

## CONCLUSION

Hospitalized patients with HDP are at a high risk of SMM. We showed that abnormal systolic blood pressure and cholesterol levels remained significantly associated with a higher risk of SMM in pregnant women with HDP and were used to develop clinical risk prediction model with high specificity and sensitivity. Our predictive model may help control the occurrence and development of severe maternal events and reduce maternal mortality.

## Data availability:

The data were anonymized, and no patient information was included to preserve confidentiality. All data used to reach the aforementioned conclusions are available for scientific purposes if needed. Please contact Dr. Xiuming Jiang.

### Authors’ Contribution:

**YB and SZ** conceptualized this study.

**XL** acquired data.

**XJ** responsible for the accuracy of the study.

**XL and XJ** designed the analyses and performed the analyses.

**YB and SZ** drafted the manuscript.

All authors read and approved the final draft of the manuscript.
